# Supplementary figures and images for: Genome-wide identification of mitogen-activated protein kinase (MAPK) cascade and expression profiling of CmMAPKs in melon (Cucumis melo L.)
Source: PLoS One. 2020 May 14;15(5):e0232756. doi: 10.1371/journal.pone.0232756 (PMC7224490; doi:10.1371/journal.pone.0232756)

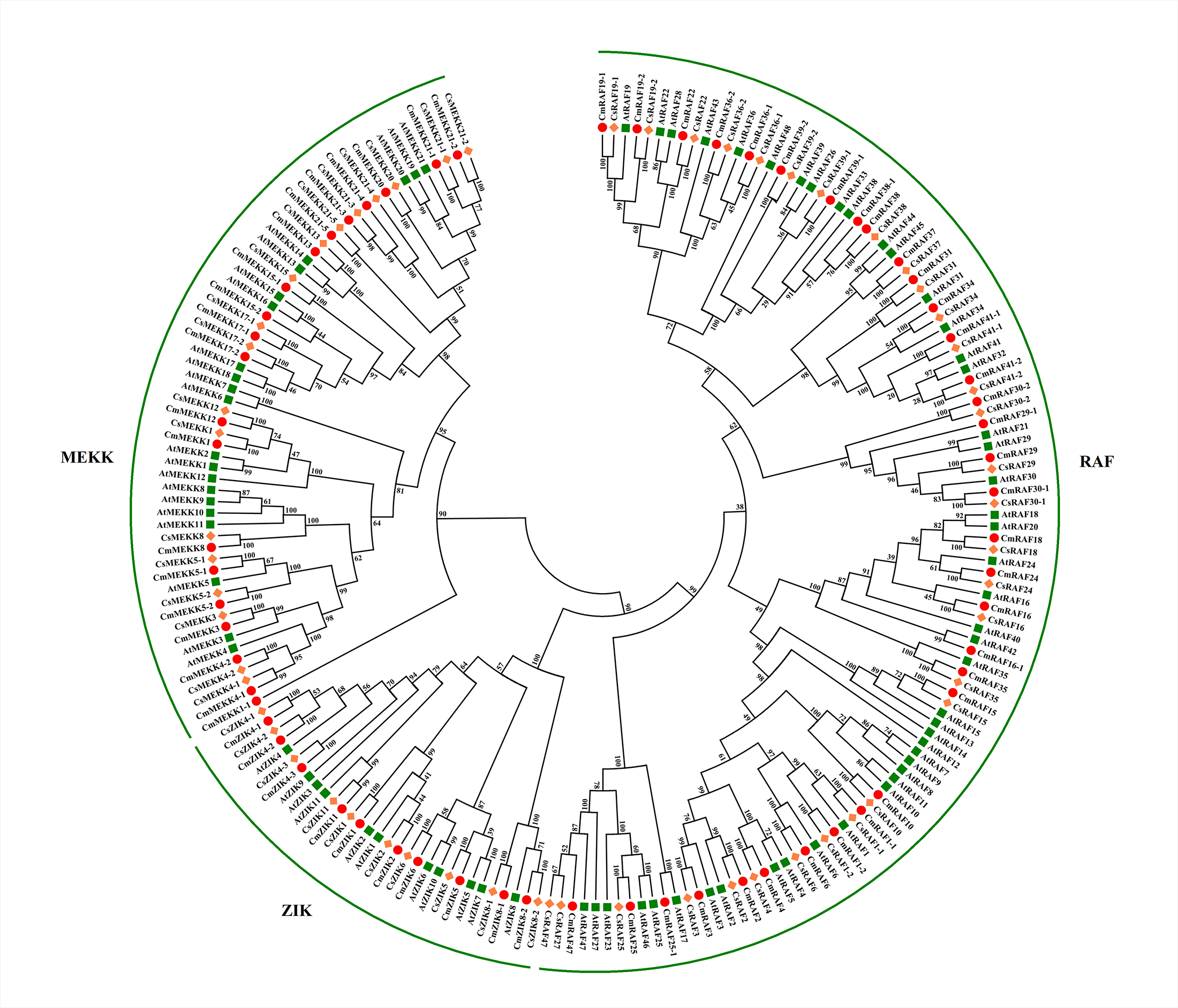

Supplement: S1 Fig — The unrooted tree was constructed, using the MEGA6.0 program, by the NJ method. Bootstrap values were calculated for 1000 replicates. (TIF) [file pone.0232756.s001.tif]

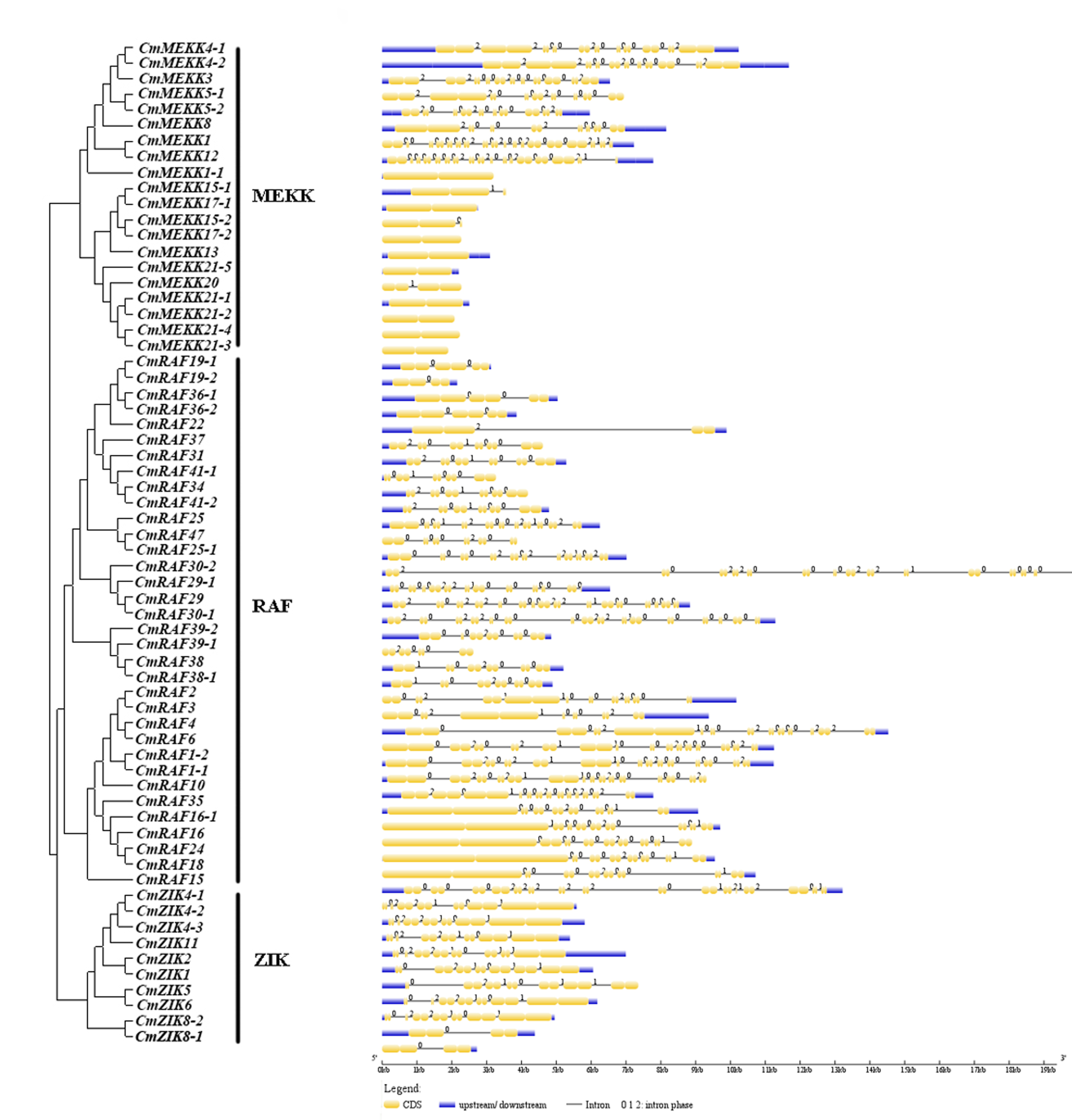

Supplement: S2 Fig — Exons and introns are shown as yellow boxes and thin lines respectively. (TIF) [file pone.0232756.s002.tif]

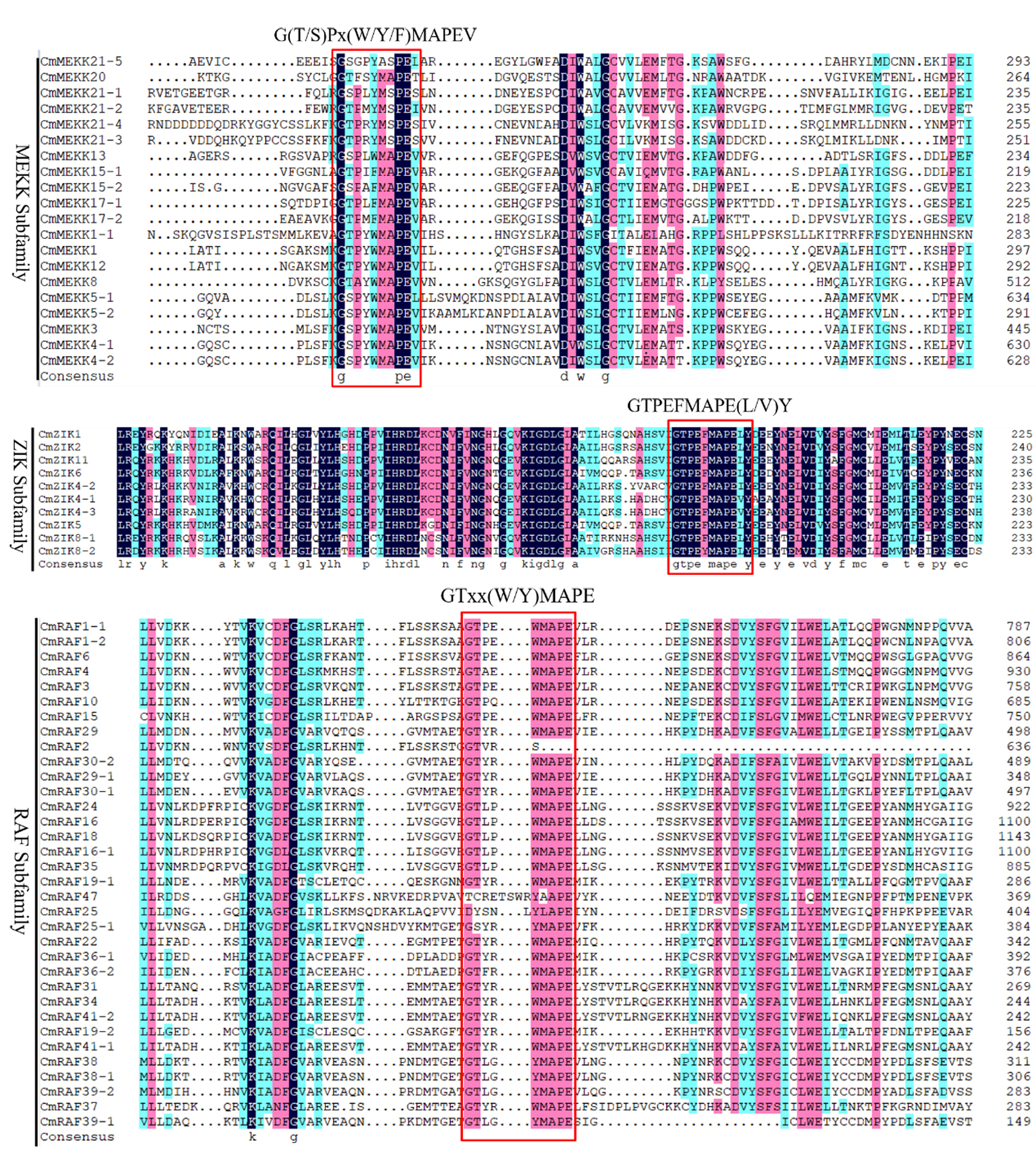

Supplement: S3 Fig — Conserved domains in MAPKKK proteins are marked with a red box and indicated. (TIF) [file pone.0232756.s003.tif]

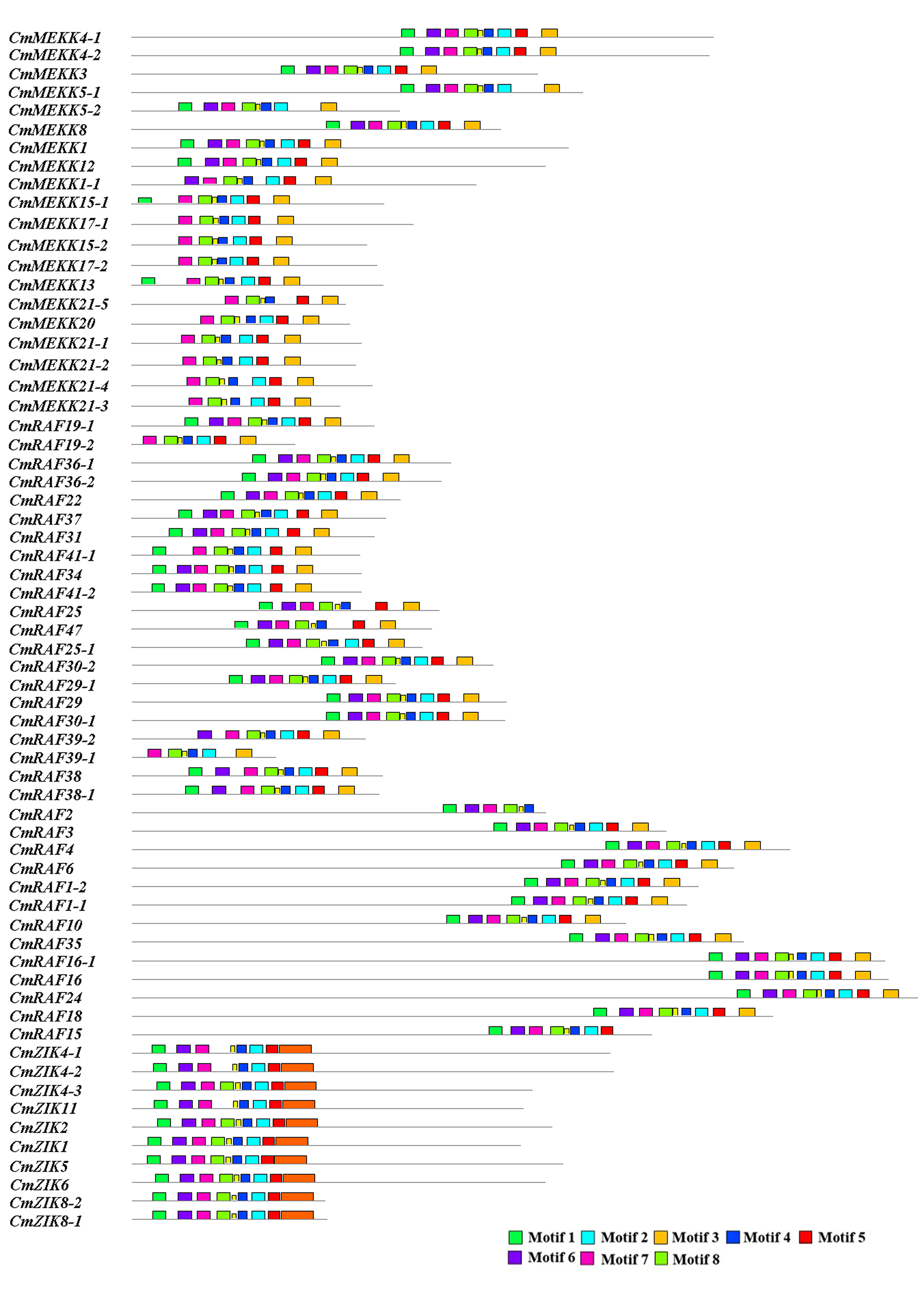

Supplement: S4 Fig — Motifs were determined using MEME program. Grey lines represent the non-conserved sequence, and each motif is indicated by a colored box numbered at the bottom. (TIF) [file pone.0232756.s004.tif]

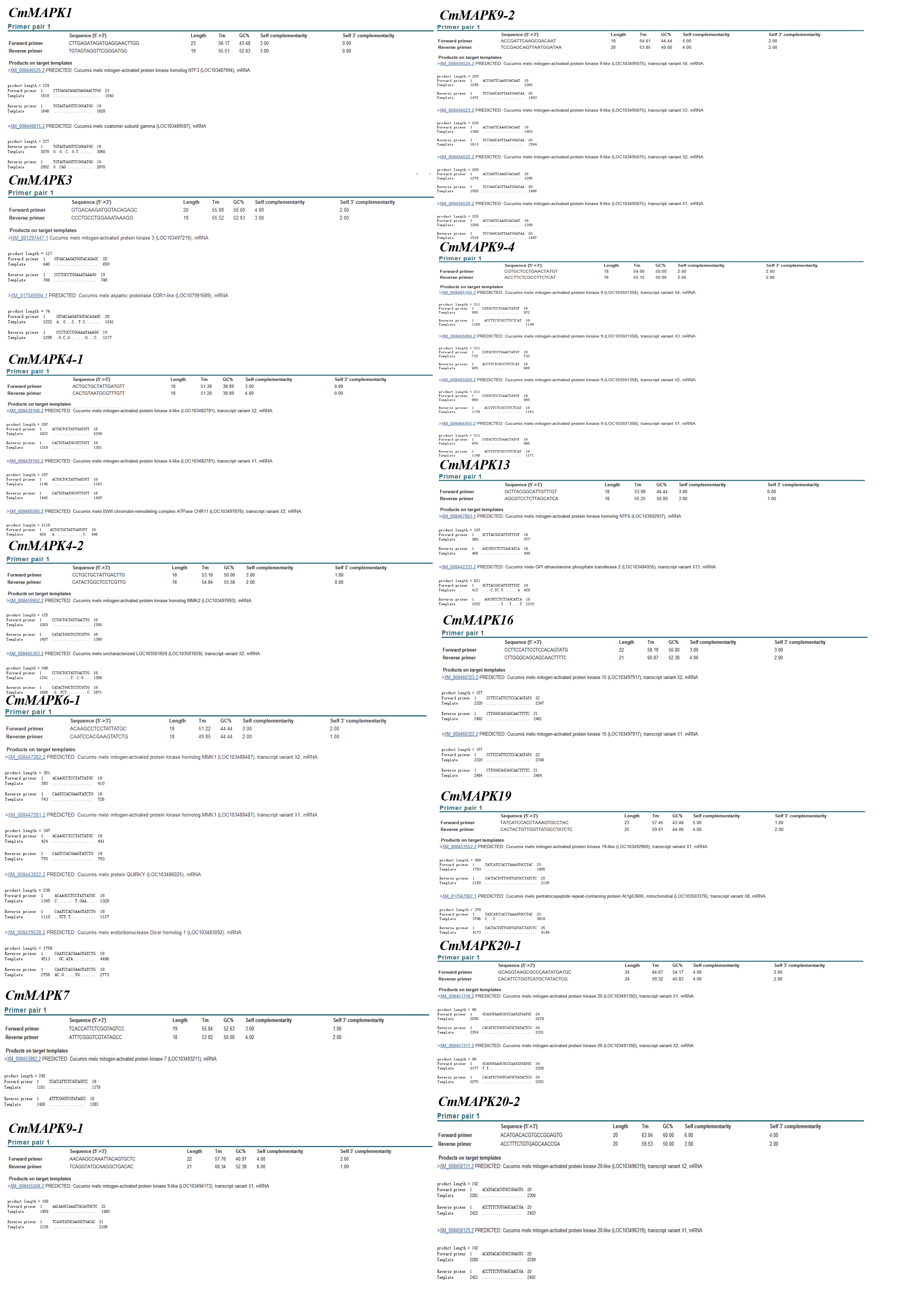

Supplement: S5 Fig — (TIF) [file pone.0232756.s005.tif]
