# Supplementary material for: Genome-wide identification of mitogen-activated protein kinase (MAPK) cascade and expression profiling of CmMAPKs in melon (Cucumis melo L.)
Source: PLoS One. 2020 May 14;15(5):e0232756. doi: 10.1371/journal.pone.0232756 (PMC7224490; doi:10.1371/journal.pone.0232756)
Supplement: S1 Table — (DOCX) [file pone.0232756.s006.docx]

**Table S1** **Summary of *CmMAPKKK* genes in melon.**

| **Gene family** | **Genes** | **protein length(aa)** | **MW** | **pI** | **Gene ID** | **Chromosome** | **Location** | **Direction** | **Subcellular location** |
| --- | --- | --- | --- | --- | --- | --- | --- | --- | --- |
|  |  |  | **(KDa)** |  |  |  |  |  |  |
| MAPKKK | *CmMEKK1* | 672 | 74.1 | 5.47 | MELO3C004269 | 5 | 24907363.. 24912573 | - | Nuclear |
|  | *CmMEKK1-1* | 529 | 58.9 | 5.78 | MELO3C006454 | 6 | 3346780.. 3348390 | - | Cytoplasm/Nuclear |
|  | *CmMEKK3* | 625 | 67.6 | 9.12 | MELO3C008058 | 8 | 729716.. 7301823 | + | Nuclear |
|  | *CmMEKK4-1* | 895 | 96.7 | 9.41 | MELO3C006925 | 6 | 7361503.. 7369050 | - | Nuclear |
|  | *CmMEKK4-2* | 889 | 95.9 | 9.35 | MELO3C005481 | 9 | 20746127.. 20752989 | + | Nuclear |
|  | *CmMEKK5-1* | 694 | 75.2 | 9.00 | MELO3C007543 | 8 | 3506011.. 3510867 | + | Nuclear |
|  | *CmMEKK5-2* | 413 | 45.7 | 7.68 | MELO3C010896 | 3 | 27864177.. 27868221 | + | Cytoplasm/Mitochondrial/Nuclear |
|  | *CmMEKK8* | 568 | 62.9 | 5.76 | MELO3C022777 | 9 | 11404329.. 11410004 | - | Nuclear |
|  | *CmMEKK12* | 637 | 70.2 | 6.43 | MELO3C014496 | 5 | 1904870.. 1910161 | + | Nuclear |
|  | *CmMEKK13* | 388 | 42.6 | 4.63 | MELO3C020535 | 12 | 623856.. 625403 | - | Nuclear/Chloroplast/Cytoplasmic |
|  | *CmMEKK15-1* | 389 | 43.2 | 5.30 | MELO3C007407 | 8 | 2584334.. 2586308 | - | Cytoplasm/Nuclear/Chloroplast |
|  | *CmMEKK15-2* | 362 | 39.8 | 4.81 | MELO3C013150 | 1 | 12589007.. 12590214 | + | Chloroplast |
|  | *CmMEKK17-1* | 434 | 48.8 | 5.29 | MELO3C019290 | 11 | 10845741.. 10847114 | - | Cytoplasm/Chloroplast |
|  | *CmMEKK17-2* | 378 | 42.6 | 4.65 | MELO3C018825 | 1 | 3143413.. 3144549 | - | Chloroplast/Cytoplasm |
|  | *CmMEKK20* | 336 | 37.3 | 8.99 | MELO3C023489 | 1 | 32815047.. 32816313 | - | Mitochondrial |
|  | *CmMEKK21-1* | 354 | 39.7 | 5.06 | MELO3C007565 | 8 | 3708647.. 3709897 | + | Cytoplasm/Chloroplast |
|  | *CmMEKK21-2* | 346 | 38.1 | 5.15 | MELO3C020859 | 11 | 3449126.. 3450166 | - | Chloroplast |
|  | *CmMEKK21-3* | 317 | 36.4 | 6.35 | MELO3C027320 | 0 | 7288883.. 7289833 | + | Nuclear/Cytoplasm |
|  | *CmMEKK21-4* | 371 | 42.9 | 9.26 | MELO3C018605 | 1 | 1466518.. 1467633 | - | Nuclear |
|  | *CmMEKK21-5* | 330 | 37.8 | 8.96 | MELO3C009916 | 4 | 26965371.. 26966470 | - | Plasma Membrane/ |
|  |  |  |  |  |  |  |  |  | Mitochondrial/Nuclear |
|  | *CmZIK1* | 597 | 69.0 | 5.27 | MELO3C025211 | 8 | 26336884.. 26342443 | + | Nuclear |
|  | *CmZIK2* | 645 | 73.0 | 6.34 | MELO3C026875 | 11 | 21026252.. 21030369 | - | Nuclear |
|  | *CmZIK4-1* | 734 | 83.7 | 5.33 | MELO3C004361 | 5 | 25871489.. 25874866 | - | Nuclear |
|  | *CmZIK4-2* | 740 | 84.2 | 4.85 | MELO3C012645 | 1 | 20745196.. 20748787 | + | Nuclear |
|  | *CmZIK4-3* | 615 | 70.9 | 5.86 | MELO3C024678 | 2 | 20211586.. 20215129 | + | Nuclear |
|  | *CmZIK5* | 662 | 74.2 | 5.48 | MELO3C006004 | 6 | 506695.. 510883 | - | Nuclear/Cytoplasm |
|  | *CmZIK6* | 635 | 71.5 | 5.13 | MELO3C005777 | 9 | 23028147.. 23031190 | + | Nuclear |
|  | *CmZIK8-1* | 299 | 34.1 | 5.77 | MELO3C014138 | 6 | 31339461.. 31341282 | - | Nuclear |
|  | *CmZIK8-2* | 297 | 33.9 | 5.13 | MELO3C025989 | 11 | 12893824.. 12897315 | - | Cytoplasm |
|  | *CmZIK11* | 601 | 68.0 | 5.02 | MELO3C002302 | 12 | 23868459.. 23873648 | + | Nuclear |
|  | *CmRAF1-1* | 852 | 94.5 | 5.62 | MELO3C024518 | 8 | 8492985.. 8499739 | - | Nuclear/Cytoplasm |
|  | *CmRAF1-2* | 870 | 96.1 | 5.65 | MELO3C009433 | 4 | 30997441.. 31006069 | - | Nuclear |
|  | *CmRAF2* | 636 | 70.3 | 6.57 | MELO3C015389 | 2 | 1269615.. 1277873 | - | Nuclear |
|  | *CmRAF3* | 683 | 76.1 | 5.15 | MELO3C016868 | 7 | 1525607.. 1532931 | + | Cytoplasm/Nuclear |
|  | *CmRAF4* | 1011 | 110.1 | 5.47 | MELO3C002125 | 12 | 24986317.. 24997810 | - | Nuclear |
|  | *CmRAF6* | 924 | 102.9 | 6.42 | MELO3C003518 | 4 | 1935422.. 1943896 | + | Nuclear/Cytoplasm |
|  | *CmRAF10* | 758 | 83.6 | 7.40 | MELO3C021267 | 11 | 27905107.. 27910614 | + | Nuclear/Cytoplasm |
|  | *CmRAF15* | 798 | 89.8 | 6.03 | MELO3C013571 | 11 | 17196141.. 17206959 | - | Nuclear |
|  | *CmRAF16* | 1162 | 130.0 | 5.57 | MELO3C006335 | 6 | 2625622.. 2631031 | - | Nuclear |
|  | *CmRAF16-1* | 1157 | 126.9 | 5.19 | MELO3C010263 | 2 | 15218767.. 15224997 | - | Nuclear |
|  | *CmRAF18* | 1208 | 133.5 | 5.27 | MELO3C019637 | 11 | 22049306.. 22055230 | + | Nuclear |
|  | *CmRAF19-1* | 373 | 42.5 | 9.07 | MELO3C007427 | 8 | 2734712.. 2736715 | + | Nuclear |
|  | *CmRAF19-2* | 251 | 28.4 | 8.68 | MELO3C002261 | 12 | 24118139.. 24119538 | - | Extracellular/Plasma Membrane |
|  | *CmRAF22* | 413 | 46.7 | 6.90 | MELO3C003908 | 5 | 19383478.. 19392121 | + | Cytoplasm |
|  | *CmRAF24* | 984 | 108.6 | 4.99 | MELO3C002407 | 12 | 23072761.. 23080525 | - | Nuclear |
|  | *CmRAF25* | 473 | 53.5 | 8.99 | MELO3C018698 | 1 | 2206846.. 2211673 | - | Mitochondrial/Nuclear |
|  | *CmRAF25-1* | 446 | 50.0 | 8.45 | MELO3C012125 | 10 | 2508620.. 2514295 | + | Mitochondrial |
|  | *CmRAF29* | 575 | 65.4 | 6.34 | MELO3C013739 | 6 | 35564980.. 35572088 | + | Cytoplasm/Nuclear |
|  | *CmRAF29-1* | 404 | 46.1 | 7.00 | MELO3C003722 | 4 | 3575562.. 3581217 | - | Cytoplasm |
|  | *CmRAF30-1* | 572 | 64.9 | 5.65 | MELO3C013322 | 1 | 15477768.. 15487337 | + | Cytoplasm |
|  | *CmRAF30-2* | 555 | 62.4 | 5.37 | MELO3C008367 | 3 | 4562292.. 4581661 | - | Cytoplasm |
|  | *CmRAF31* | 373 | 42.0 | 9.23 | MELO3C009110 | 4 | 32962731.. 32966893 | + | Cytoplasm/Nuclear |
|  | *CmRAF34* | 353 | 39.8 | 7.16 | MELO3C026256 | 2 | 25570643.. 25573768 | - | Cytoplasm |
|  | *CmRAF35* | 939 | 105.1 | 5.95 | MELO3C004081 | 5 | 22742971.. 22749223 | - | Nuclear |
|  | *CmRAF36-1* | 491 | 55.7 | 9.31 | MELO3C007280 | 8 | 1916345.. 1919904 | + | Mitochondrial |
|  | *CmRAF36-2* | 476 | 54.2 | 9.40 | MELO3C002609 | 12 | 21601873.. 21604301 | + | Mitochondrial |
|  | *CmRAF37* | 391 | 44.3 | 5.13 | MELO3C007810 | 8 | 5520190.. 5523629 | + | Nuclear/Cytoplasm |
|  | *CmRAF38* | 384 | 42.2 | 7.06 | MELO3C009953 | 4 | 26594557.. 26598607 | + | Cytoplasm/Nuclear |
|  | *CmRAF38-1* | 379 | 42.3 | 7.94 | MELO3C017099 | 2 | 25092065.. 25095816 | - | Cytoplasm |
|  | *CmRAF39-1* | 221 | 24.6 | 8.65 | MELO3C015795 | 1 | 28880422.. 28882376 | - | Mitochondrial/Nuclear |
|  | *CmRAF39-2* | 358 | 40.1 | 8.79 | MELO3C006511 | 6 | 3840870.. 3844648 | + | Cytoplasm |
|  | *CmRAF41-1* | 299 | 33.6 | 8.49 | MELO3C026587 | 4 | 26072313.. 26075612 | - | Mitochondrial/Nuclear/Cytoplasm |
|  | *CmRAF41-2* | 353 | 39.3 | 6.80 | MELO3C021586 | 9 | 4170353.. 4174081 | + | Cytoplasm |
|  | *CmRAF47* | 267 | 29.8 | 4.98 | MELO3C019698 | 11 | 23272282.. 23287335 | - | Cytoplasm |
