# Supplementary material for: Genome-wide identification of mitogen-activated protein kinase (MAPK) cascade and expression profiling of CmMAPKs in melon (Cucumis melo L.)
Source: PLoS One. 2020 May 14;15(5):e0232756. doi: 10.1371/journal.pone.0232756 (PMC7224490; doi:10.1371/journal.pone.0232756)
Supplement: S2 Table — (DOCX) [file pone.0232756.s007.docx]

**Table S2** **Primers used in this study.**

| **Primer name** | **Sequence(5’-3’)** |
| --- | --- |
| *CmMAPK1* | F:CTTGAGATAGATGAGGAACTTGG  R:TGTAGTAGGTTCGGGATGG |
| *CmMAPK3* | F:GTGACAAGATGGTACAGAGC  R:CCCTGCCTGGAAATAAAGG |
| *CmMAPK4-1* | F:ACTGCTGCTATTGATGTT  R:CACTGTAATGCGTTTGTT |
| *CmMAPK4-2* | F:CCTGCTGCTATTGACTTG  R:CATACTGGCTCCTCGTTG |
| *CmMAPK6-1* | F:ACAAGCCTCCTATTATGC  R:CAATCCACGAAGTATCTG |
| *CmMAPK7* | F:TCACCATTCTCGGTAGTCC  R:ATTTCGGGTCGTATAGCC |
| *CmMAPK9-1* | F:AACAAGCCAAATTACAGTGCTC  R:TCAGGTATGCAAGGCTGACAC |
| *CmMAPK9-2* | F:ACCGATTCAAGCGACAAT  R:TCCGAGCAGTTAATGGATAA |
| *CmMAPK9-4* | F:CGTGCTCCTGAACTATGT  R:ACCTTCTCGCCTTCTCAT |
| *CmMAPK13* | F:GCTTACGGCATTGTTTGT  R:AGCGTCCTCTTAGCATCA |
| *CmMAPK16* | F:CCTTCCATTCCTCCACAGTATG  R:CTTGGGCAGCAGCAACTTTTC |
| *CmMAPK19* | F:TATCATCCACCTAAAGTGCCTAC  R:CACTACTGTTGGTTATGCCTATCTC |
| *CmMAPK20-1* | F:GCAGGTAAGCGCCCAATATGATGC  R:CACATTCTGGTCATGCTATACTCG |
| *CmMAPK20-2* | F:ACATGACACGTGCCGGAGTG  R:ACCTTTCTGTGAGCAACCGA |
| *18s rRNA* | F:GTGATGGTGTGAGTCACACTGTTC  R:ACGACCAGCAAGGTCCAAAC |
