# Supplementary material for: Genome-wide identification of mitogen-activated protein kinase (MAPK) cascade and expression profiling of CmMAPKs in melon (Cucumis melo L.)
Source: PLoS One. 2020 May 14;15(5):e0232756. doi: 10.1371/journal.pone.0232756 (PMC7224490; doi:10.1371/journal.pone.0232756)
Supplement: Attachment — Submitted filename: Response to Reviewers.docx [file pone.0232756.s008.docx]

From: "PLOS ONE" <em@editorialmanager.com>
Date: 2020-03-06 21:53:08
To: "Hongyan Qi" <[hyqiaaa@126.com](mailto:hyqiaaa@126.com)>
Subject: PLOS ONE Decision: Revision required [PONE-D-20-01288] - [EMID:1cb24cf0edede256]

PONE-D-20-01288
Genome-wide identification of mitogen-activated protein kinase (MAPK) cascade and expression profiling of *CmMAPKs* in melon (*Cucumis melo* L.)
PLOS ONE

Dear Dr Qi,

Thank you for submitting your manuscript to PLOS ONE. After careful consideration, we feel that it has merit but does not fully meet PLOS ONE’s publication criteria as it currently stands. Therefore, we invite you to submit a revised version of the manuscript that addresses the points raised during the review process.


We would appreciate receiving your revised manuscript by Apr 20 2020 11:59PM. When you are ready to submit your revision, log on to <https://www.editorialmanager.com/pone/> and select the 'Submissions Needing Revision' folder to locate your manuscript file.

If you would like to make changes to your financial disclosure, please include your updated statement in your cover letter.

To enhance the reproducibility of your results, we recommend that if applicable you deposit your laboratory protocols in protocols.io, where a protocol can be assigned its own identifier (DOI) such that it can be cited independently in the future. For instructions see: <http://journals.plos.org/plosone/s/submission-guidelines#loc-laboratory-protocols>

Please include the following items when submitting your revised manuscript:

- A rebuttal letter that responds to each point raised by the academic editor and reviewer(s). This letter should be uploaded as separate file and labeled 'Response to Reviewers'.
- A marked-up copy of your manuscript that highlights changes made to the original version. This file should be uploaded as separate file and labeled 'Revised Manuscript with Track Changes'.
- An unmarked version of your revised paper without tracked changes. This file should be uploaded as separate file and labeled 'Manuscript'.

Please note while forming your response, if your article is accepted, you may have the opportunity to make the peer review history publicly available. The record will include editor decision letters (with reviews) and your responses to reviewer comments. If eligible, we will contact you to opt in or out.

We look forward to receiving your revised manuscript.

Kind regards,

Yuan Huang
Academic Editor
PLOS ONE

Re: Thank you for your letter and the comments concerning our manuscript entitled “Genome-wide identification of mitogen-activated protein kinase (MAPK) cascade and expression profiling of *CmMAPKs* in melon (*Cucumis melo* L.)”. The comments have been carefully took into account and we have made a careful revision on the original manuscript. All revised portions are marked in red in the revised manuscript. We have carefully addressed the issues in view letter point-by-point in the revised manuscript.

Journal Requirements:

When submitting your revision, we need you to address these additional requirements:

1. Please ensure that your manuscript meets PLOS ONE's style requirements, including those for file naming. The PLOS ONE style templates can be found at <http://www.plosone.org/attachments/PLOSOne_formatting_sample_main_body.pdf> and <http://www.plosone.org/attachments/PLOSOne_formatting_sample_title_authors_affiliations.pdf>

 Re: Thank you for your suggestion. We ensured that our manuscript meets PLOS ONE's style requirements.

2. PLOS requires an ORCID iD for the corresponding author in Editorial Manager on papers submitted after December 6th, 2016. Please ensure that you have an ORCID iD and that it is validated in Editorial Manager. To do this, go to ‘Update my Information’ (in the upper left-hand corner of the main menu), and click on the Fetch/Validate link next to the ORCID field. This will take you to the ORCID site and allow you to create a new iD or authenticate a pre-existing iD in Editorial Manager. Please see the following video for instructions on linking an ORCID iD to your Editorial Manager account: <https://www.youtube.com/watch?v=_xcclfuvtxQ>

 Re: Thank you for your suggestion. We created a new ORCID iD (0000-0002-7592-8749) in Editorial Manager.

3. Please upload a copy of Supporting Information Table S4 which you refer to in your text on page 8.

Re: We are sorry for our incorrect expression. Actually, Table S4 which we refer to in our text on page 8 is Table S2. Please see in the line 153 of the manuscript revision.

[Note: HTML markup is below. Please do not edit.]

Reviewers' comments:

Reviewer's Responses to Questions

**Comments to the Author**

5. Review Comments to the Author

Please use the space provided to explain your answers to the questions above. You may also include additional comments for the author, including concerns about dual publication, research ethics, or publication ethics. (Please upload your review as an attachment if it exceeds 20,000 characters)

Reviewer #1: This manuscript describes the different expression patterns of MAPK genes under virous of treatments. There some concerns are:
1. The authors are recommended to ask help from English native speakers.

Re: We have followed your suggestion. The English native speakers have helped us to check and revised our manuscript, please see the file of “Revised Manuscript with Track Changes”.

2. Please check the website of database of cucumber and watermelon.

Re: We have followed your suggestion and revised the website. Please see in the line 96-97 of the manuscript revision.

3. exhibit chromosomal location based on the physical position.

Re: We have followed your suggestion and reformatted the Figure 5. The physical position was exhibited on the left in the Figure 5. Please see the revised figure in the figures file.

4. The expression pattern of genes response to different treatments is different, it may be better to separately exhibit the results of different treatments.

Re: We have followed your suggestion and reformatted the Figure 6, 7 and 9. Please see the revised figures in the figures file.

5. the results of Expression profiles of *CmMAPKs* in response to pathogen infection are not clearly presented. And the conclusion, it is speculated that *CmMAPKs* may play a negative regulatory role in the mechanism of disease resistance, is not precise.

Re: We are sorry for our inaccurate expression. We have revised the sentences. Please see in the line 337-345 of the manuscript revision.

6. In the Discussion section, the findings should be compared with the findings in prior studies, but findings in this study and that in in prior studies should not exhibit in separate paragraphs.

Re: We have followed your suggestion and organized the language representations exhibiting our findings and that in prior studies in one paragraph. Please see in the line 382-434 of the manuscript revision.

Reviewer #2: Genome-wide identification and expression profiling of MAPK genes in melon were Investigated in this study, which will provide some information to the related research community. The following problems, not limiting to these, should be taken into consideration to improve the robustness of the research and the quality of manuscript.
1. Biological replication should be adopted in the experiments.

Re: We are sorry for our incorrect expression. Three biological and three technical replicates were adopted in the experiments mentioned in our manuscript. We have revised it. Please see in the line 154-155 of the manuscript revision.

2. In qRT-PCR analysis, several reference genes have been validated in melon, which are better than 18s rRNA. The authors should use the validated reference genes.

Re: We have considered your suggestion. Please allow me to explain that the reference gene we used is more stable than actin after years of our laboratory verification and has been used in many experiments. There are several papers we published using 18s rRNA (Yufan Tang et al., 2015; Yazhong Jin et al., 2016; Xiaoou Guo et al., 2017; Chong Zhang et al., 2017; Qiaojuan Xing et al., 2019).

3. Family genes have high sequence similarities and share several conserved motifs among them. As a result, specificity of primers is vital for the success of qRT-PCR analysis. The authors should give the detailed information on the method of primer design and provide the related supporting figures.

Re: We have followed your suggestion. The method of primer design is according to the reference below (Jian Yao et al., 2015). Then we uploaded the primers to NCBI website (<https://www.ncbi.nlm.nih.gov/tools/primer-blast/>) to check specificity of primers. Please see the related supporting information in S5 figure.

4. In the section of statistical analysis, the method used is not correct.

 Re: We are sorry for our incorrect expression. In the section of statistical analysis, significant differences were compared using a one-way ANOVA following Duncan’s multiple range test at p<0.05 level except expression profiles of *CmMAPKs* under RL induction were conducted by t test at p<0.05 level. We have revised it. Please see in the line 157-160 of the manuscript revision.

6. PLOS authors have the option to publish the peer review history of their article ([what does this mean?](https://journals.plos.org/plosone/s/editorial-and-peer-review-process#loc-peer-review-history)). If published, this will include your full peer review and any attached files.


If you choose “no”, your identity will remain anonymous but your review may still be made public.


**Do you want your identity to be public for this peer review?** For information about this choice, including consent withdrawal, please see our [Privacy Policy](https://www.plos.org/privacy-policy).

Reviewer #1: No

Reviewer #2: No


[NOTE: If reviewer comments were submitted as an attachment file, they will be attached to this email and accessible via the submission site. Please log into your account, locate the manuscript record, and check for the action link "View Attachments". If this link does not appear, there are no attachment files to be viewed.]


While revising your submission, please upload your figure files to the Preflight Analysis and Conversion Engine (PACE) digital diagnostic tool, <https://pacev2.apexcovantage.com/>. PACE helps ensure that figures meet PLOS requirements. To use PACE, you must first register as a user. Registration is free. Then, login and navigate to the UPLOAD tab, where you will find detailed instructions on how to use the tool. If you encounter any issues or have any questions when using PACE, please email us at [figures@plos.org](mailto:figures@plos.org). Please note that Supporting Information files do not need this step.

References:

1. Tang TF, Zhang C, Cao SX, Wang X, Qi HY. The Effect of *CmLOXs* on the Production of Volatile Organic Compounds in Four Aroma Types of Melon (Cucumis melo). PLOS ONE. 2015; 10(11). doi:10.1371/journal.pone.0143567
2. Jin YZ, Zhang C, Liu W, Tang YF, Qi HY, Chen H, et al. The Alcohol Dehydrogenase Gene Family in Melon (*Cucumis melo* L.): Bioinformatic Analysis and Expression Patterns. Frontiers in Plant Science.2016;7. doi:10.3389/fpls.2016.00670
3. Guo XO, Xu JJ, Cui XH, Chen H, Qi HY. iTRAQ-based Protein Profiling and Fruit Quality Changes at Different Development Stages of Oriental Melon.BMC Plant Biology.2017;17. doi:10.1186/s12870-017-0977-7
4. Zhang C, Cao SX, Jin YZ, Ju LJ, Chen Q, Xing QJ, et al. Melon13-lipoxygenase *CmLOX18* may involved in C6 volatiles biosynthesis in fruit. Scientific Reports.2017;7. doi:10.1038/s41598-017-02559-6
5. Xing QJ, Zhang XL, Li YP, Shao QX, Cao SX, Wang F, et al. The lipoxygenase *CmLOX13* from oriental melon enhanced severe drought tolerance via regulating ABA accumulation and stomatal closure in Arabidopsis. Environmental and Experimental Botany.2019; 103815.doi:10.1016/j.envexpbot.2019.103815
6. Yao J, Dong ZH, Ma XX, Wang XF. A simple method of real-time PCR primer design based on free online software. Genomics and Applied Biology. 2015; 34(12):2779-2784. doi:10.13417/j.gab.034.002779
